# A New Penicillin?

**DOI:** 10.3390/antibiotics9030117

**Published:** 2020-03-11

**Authors:** Mark Wainwright

**Affiliations:** School of Pharmacy & Biomolecular Sciences, Liverpool John Moores University, Byrom Street, Liverpool L3 3AF, UK; mark_wainwright@hotmail.com

**Keywords:** antimicrobial drug resistance, infection control, infectious disease prophylaxis, methylene blue, photoantimicrobial, photodecolonisation

## Abstract

The spectre of antimicrobial resistance looms very large indeed in the 21st century; the supply of efficacious conventional drugs is short and not guaranteed, for various reasons. It is time to look elsewhere for answers and for protocols which might be used in tandem with our diminishing arsenal in order to protect vital drugs. This could bridge the gap before new development in conventional antimicrobial therapy occurs, or might be a longer-term solution, particularly in the area of infectious disease prophylaxis (conventional-sensitive or -resistant). Reliable and safe protocols have been developed for the use of photoantimicrobials in this respect, offering much greater coverage, in terms of the microbial target, than Fleming ever imagined.

## 1. The Age of Resistance

Among the furore concerning our problems with increasing antimicrobial resistance (AMR), there is a thread, usually termed ’The rise of the Superbug’ or something similar, which appears to be regarded as some sort of highly organised, separatist political movement, rather than the organic expression of evolution that it is. Selective pressure as an evolutionary driver is well understood, so there should be no need to lionise the result.

However, given that microbes capable of dealing with our current antimicrobial armoury with such efficiency exist, what should we do about them? Admittedly, there are initiatives aimed at countering the problem and pronouncements by those governing healthcare, i.e., The Chief Medical Officer and Lord O’Neill in the UK, and the Centers for Disease Control and Prevention (CDC), UN, and WHO on the broader stage [1]. However, this is recognised as a complex problem requiring much more than scientific endeavours for its solution.

It is established that we should decrease the exposure of the microbiome to antimicrobial drugs by prescribing only when necessary, whether for human or animal diseases, with bad examples including the prescription of antibiotics for viral illnesses and antibiotic use in agricultural growth promotion. Equally, the global distribution of drugs must be addressed so that self-medication via uncontrolled supply might also be stopped.

Clearly, there are difficult – and in some cases, perhaps impossible – tasks to be addressed, but in one area, i.e., the development of new antimicrobial agents, there should be clarity and a concentration of efforts.

When Alexander Fleming made reference to bacterial drug resistance in his Nobel Prize acceptance speech [2], he was reporting laboratory and clinical findings, rather than providing the underlying mechanisms, as these were as yet undiscovered. The penicillins in use at the time, V and G, would have presented β-lactamase-type capabilities. In later years, this would include penicillin-binding proteins and extended-spectrum β-lactamases, but each of these mechanisms is successful—as is the case with resistance mechanisms against other conventional antimicrobials—because of the single mode/single site of antimicrobial action approach which has predominated throughout the ‘antibiotic era’.

## 2. Fighting Resistance

The great danger in constructing a response to global drug resistance is that we are attempting to broaden our current antimicrobial arsenal using the same approach, i.e., restricted usage to slow the onset. New biomolecular targets are, of course, desirable, but these are only likely to provide longevity if they offer multiplicity, preferably in both the target site and the mode of attack of the resulting drug candidates. Clearly, this is not a simple or inexpensive undertaking.

However, there is a further point to consider, i.e., is there a general wish to fight the drug-resistance threat scientifically, or rather, merely to discover/develop new antimicrobial drugs of the same type, to be employed in the same way? Most scientific reviews and media coverage seem to imply the latter, with the majority of articles/programmes covering the search for replacement antibiotics, such as teixobactin [3], and only a tiny fraction being dedicated to other methods, usually biological, such as vaccination or the use of bacteriophages [4]. The search for these new replacement agents has the same rationale as that of 70 years earlier, i.e., compounds produced by microorganisms for chemical defence against other microorganisms, only in different areas of the globe, or using alternative culturing methods (e.g., teixobactin). Given that we have been spoiled by the ease with which conventional antimicrobial agents are administered, as well as their general availability, it is unsurprising that maintaining the status quo is so desirable. Antimicrobial stewardship clearly means a departure from this situation, and given support (and cooperation) from all sides, can at least cause some arrest in the development of resistant microbes [5].

It is clear that several pharmaceutical houses have withdrawn from the fight, usually citing the enormous costs involved in developing new drugs and the relative paucity of return, which puts greater pressure on those that remain. This should be seen as an opportunity for others (including public funders), but will only be attractive to the commercial sector if the cost basis is made more manageable.

## 3. Alternative Approaches

However, there are less expensive approaches which would involve agents which are already available and approved by relevant regulatory bodies. The antiseptics, such as the quaternary ammonium compounds (QACs), and related bisbiguanides, such as the chlorhexidine salts, are well known and have been in use since before the Second World War. Similarly, dye-based therapeutics such as the flavines and methylene blue were widely used anti-infectives until the middle of the 20th century.

The modern scientist might consider these compounds to be crude and old fashioned. However, the situation in 21st-century infection control is worsening rapidly, with nightmare figures predicted for the numbers of deaths due to resistant microbial infection (10 million per year by 2050, according to the O’Neill Report). In recent years, there have been various published studies under the heading of ‘Old Drugs for New Bugs’, which usually show recovered activity, e.g., for tetracyclines, aminoglycosides, etc., against relevant pathogens implicated in AMR as a result of the drugs’ original withdrawal implemented several years before. Such studies normally reflect the down-regulation of pertinent resistance mechanisms during the intervening period.

Reintroducing, or rather repurposing, the compounds proposed above would have to involve more selective use. Given that systemic disease cannot be treated with antiseptics due to the fact that the internal concentrations required would be too high, and thus toxic for human or animal use, localised disease offers a more suitable and achievable goal. This may seem to be something of an underachievement, but provided at the right time and employed in the right way, this method may be a life-saver. This is particularly relevant with the dyes noted above, when they are used in conjunction with light activation, causing the production of reactive oxygen species (ROS) in situ.

A useful example here would be that of suspected bacterial tonsillitis. Typically, this would be treated with a short course of amoxicillin, which might or might not work, depending on the resistance profile of the (presumed) causative bacteria. Since the antibacterial formulation is orally administered, much of the flora of the alimentary canal will thus be provided with a dose sufficient to kill a fraction of the susceptible population, leaving the resistant cohort unharmed and ready to take over the space thus liberated. This selective pressure obviously increases the resistant population, but the resulting physical spread of ‘new’ bacteria into areas normally occupied by commensals can also lead to illness due to the toxins they release. An extreme instance of this is *Clostridioides difficile*-associated diarrhoea, a manifestation of bacterial overgrowth in the colon following extended broad-spectrum antibacterial use [6]. Such an outcome would be unusual following straightforward tonsillitis treatment, but upset stomachs caused by a similar mechanism, particularly in juvenile patients, are not.

Given that the intended infection in tonsillitis is localised to the oropharynx, spreading the dose of the antibacterial throughout the body like this may seem somewhat contradictory, but this has been the standard therapy for around 80 years.

There is a further problem with this approach in that most conventional antibacterial drugs act against growing and dividing cells, rather than the resting/quiescent population, which means that killing or inactivating sufficient numbers may require a period of time, rather than being immediate. Similarly, small populations of persister cells also exist in each new generation which are resistant to conventional drugs [7].

Conversely, the direct application of antiseptics to the oropharynx, as a spray, gargle, or lozenge, locally kills bacteria directly, with little or no effect on the remaining microbiome. This approach could employ either conventional antiseptics, such as benzalkonium chloride or chlorhexidine digluconate, which are employed at high concentrations and act principally via membrane and enzyme disruption [8], or a photosensitiser, such as methylene blue, activated by red light.

### 3.1. Photoantimicrobials

Of the two methods, the use of conventional antiseptics appears to offer a less complicated route, requiring no light activation. However, the production of ROS by photosensitisers offers a more efficient, rapid, and broad-spectrum kill at much lower concentrations. This is also important with respect to the pathogen class, as detailed below. Given the nonspecific modes of action entailed, both conventional antiseptics and photoantimicrobials will, of course, also inactivate viruses and yeasts, unlike conventional antibacterial drugs.

Consequently, this direct approach offers a rapid solution to bacterial or viral tonsillitis which neither uses valuable conventional antibacterial drugs nor adds to the selective pressure for resistance development among the microbiota normally associated with conventional therapy. 

For a relatively simple disease presentation, such as tonsillitis, there should be little reason against a proper introduction of this approach. The arguments against the utilisation of photosensitisers usually involve facts, e.g., that methylene blue is a coloured, staining material and it requires light activation for significant antimicrobial effects to be achieved. This is insurmountable, i.e., tissue staining is transient and the application of light to the back of the throat is no more invasive than a routine dental examination. However, it is fair to say that the employment of photoantimicrobials would represent a considerable change in anti-infective practice. The question remains whether those involved in delivery can be persuaded to make the change in view of the advantages over the conventional therapy outlined above. 

Tonsillitis is normally a simple, self-limiting illness, but it can progress to much more serious conditions, such as pneumonia, meningitis, and septicaemia [9]. Obviously, the clearance of bacterial infection at the stage of tonsillitis (or otitis media, laryngitis, etc.) is preferable, as opposed to dealing with a life-threatening disseminated disease. However, a failing battery of conventional antibacterial drugs means that such progressions will become more common, and the possibility of replacing conventional therapy at this stage with one which neither suffers from nor leads to resistance should thus be attractive.

The use of a photoantimicrobial approach for transmission inhibition is similarly attractive. Methylene blue has been in use for methicillin-resistant *Staphylococcus aureus* (MRSA) photodecolonisation for around a decade in Vancouver [10] where elective patients are routinely treated with a local application (methylene blue + red light) to the nostrils, with impressive subsequent decreases in postoperative MRSA infections. Such an approach could be used prophylactically to avoid the spread of infection among close communities (student accommodation, military establishments, and prisons), and has considerable potential against both viral (‘flu, coronavirus (COVID)) and bacterial (meningitis) outbreaks.

### 3.2. ‘Acceptable’ Alternatives?

Given that dye-based therapeutics were established before the First World War, QACs in the 1930s and photoantimicrobials a quarter of a century ago, it must be asked why these approaches are not currently employed more directly in infection control protocols. It is clear that they are not really being considered for future mainstream use, at least in affluent regions, since they are never mentioned as part of the group of ‘alternative’ approaches to fighting AMR which is now sometimes listed in government/Non-Government Organisation sources. However, since this listing is very new in such documents, perhaps it reflects a grudging admission that there is any option at all outside the conventional route. Since the alternatives usually given are biologicals, i.e., vaccines and phage therapy, this hardly constitutes scientific adventure, and may, in any case, be too specific for the general, broad-spectrum coverage of infectious disease which will be required.

## 4. A New ‘Penicillin’?

The irony of the current situation regarding the non-uptake—indeed, the non-consideration—of photoantimicrobials is, of course, lost on the huge majority of the population. This is perhaps unavoidable, given the continuing pre-eminence of the penicillin myth in popular culture and, by extension, the unassailable position of ‘antibiotics’ in modern infection control. Particularly with cationic photosensitisers, a truly antimicrobial capability in the face of a rapidly elevating resistance crisis is available. Descendants of Fleming’s mould broth, such as the 21st -century carbapenems, are now routinely nullified by resistance mechanisms developed as a result of β-lactam prophylaxis, or other misuse. Not only does cationic methylene blue kill bacteria which expresses carbapenemase capability (of any type), but it may also be used prophylactically against colonisation by such bacteria without promoting resistance development. Furthermore, protection against viral infection in this way would also both nullify rapid viral mutation rates, as seen, e.g., with COVID-19, and maintain efficacy regardless of strain and antigenic shift/drift such as between serotypes in influenza.
